# Difficult-to-treat HIV in Sweden: a cross-sectional study

**DOI:** 10.1186/s12879-024-09214-2

**Published:** 2024-03-18

**Authors:** Olof Elvstam, Viktor Dahl, Anna Weibull Wärnberg, Susanne von Stockenström, Aylin Yilmaz

**Affiliations:** 1 Department of Translational Medicine, Lund University, Malmö, Sweden; 2 Department of Infectious Diseases, Växjö Central Hospital, Växjö, Sweden; 3 Unit of Infectious Diseases/Venhälsan, Southern Hospital, Stockholm, Sweden; 4 Department of Infectious Diseases, Karolinska University Hospital, Stockholm, Sweden; 5 Gilead Sciences, Solna, Sweden; 6 Department of Infectious Diseases, Institute of Biomedicine, University of Gothenburg, Sahlgrenska Academy, Gothenburg, Sweden; 7 Department of Infectious Diseases, Sahlgrenska University Hospital, Region Västra Götaland, Gothenburg, Sweden

**Keywords:** Antiretroviral treatment, Heavily treatment experienced, HIV resistance, Patient reported outcome measures

## Abstract

**Background:**

Our aim was to examine the prevalence and characteristics of difficult-to-treat HIV in the current Swedish HIV cohort and to compare treatment outcomes between people with difficult and non-difficult-to-treat HIV.

**Methods:**

In this cross-sectional analysis of the Swedish HIV cohort, we identified all people with HIV currently in active care in 2023 from the national register InfCareHIV. We defined five categories of difficult-to-treat HIV: 1) advanced resistance, 2) four-drug regimen, 3) salvage therapy, 4) virologic failure within the past 12 months, and 5) ≥ 2 regimen switches following virologic failure since 2008. People classified as having difficult-to-treat HIV were compared with non-difficult for background characteristics as well as treatment outcomes (viral suppression and self-reported physical and psychological health).

**Results:**

Nine percent of the Swedish HIV cohort in 2023 (*n* = 8531) met at least one criterion for difficult-to-treat HIV. Most of them had ≥ 2 regimen switches (6%), and the other categories of difficult-to-treat HIV were rare (1–2% of the entire cohort). Compared with non-difficult, people with difficult-to-treat HIV were older, had an earlier first year of positive HIV test and lower CD4 counts, and were more often female. The viral suppression rate among people with difficult-to-treat HIV was 84% compared with 95% for non-difficult (*p* = 0.001). People with difficult-to-treat HIV reported worse physical (but not psychological) health, and this remained statistically significant after adjustment for age, sex, and transmission group.

**Conclusions:**

Although 9% of the HIV cohort in Sweden in 2023 were classified as having difficult-to-treat HIV, a large proportion of these were virally suppressed, and challenges such as advanced resistance and need for salvage therapy are rare in the current Swedish cohort.

## Background

Treatment with antiretroviral therapy (ART) has transformed HIV from a deadly disease into a manageable condition with survival rates close to that of HIV-negative people [1]. Viral suppression also effectively prevents onwards transmission [2]. In the latest report from the Swedish national HIV register, InfCareHIV, 99% of known people with HIV in Sweden were reported to receive ART, and 95% of those reached a viral load of < 50 copies/mL [3]; Sweden has thus reached the second and third UNAIDS 95-95-95 targets [4]. In light of this accomplishment, we wanted to explore remaining challenges related to heavily treatment-experienced (HTE) people with HIV and people with recent or repeated virologic failure, who we will collectively refer to as difficult-to-treat HIV.

The proportion of people who are HTE in Sweden is not known. In other high-income settings, around 10% have been identified as HTE [5, 6], but there are substantial differences depending on whether the definition was based on resistance profile and/or ART history. HTE people with HIV face higher risks of incomplete CD4 count recovery [5, 6]. A higher risk of AIDS and non-AIDS morbidity has also been reported [5], although an analysis of a large European cohort suggest that this could be completely explained by advanced age, low CD4 counts, and prior comorbidity [6]. Importantly, treatment experience is not the only factor that could contribute to difficult-to-treat HIV. Virologic failure, usually defined as repeated viral load measurements > 200 copies/mL [7], can develop with or without antiretroviral resistance; failure without resistance is typically caused by insufficient adherence. Since virologic failure has clear consequences for disease progression [8, 9] and requires careful clinical management, we wanted to consider the history of virologic failure – and not only treatment history – when defining difficult-to-treat HIV.

In this study, we aimed to describe the current situation of difficult-to-treat HIV in Sweden in 2023. We analyzed factors associated with having difficult-to-treat HIV and compared treatment outcomes between people classified as having difficult and non-difficult-to-treat HIV.

## Methods

We conducted a cross-sectional study using data from the Swedish national HIV registry, InfCareHIV. The Swedish HIV cohort has been described elsewhere [10]. In brief, care is provided at 29 infectious disease clinics across the country and ART is provided free of charge. InfCareHIV contains clinical and demographic data on > 99% of all people with diagnosed HIV in Sweden. The register was founded in 2003 and achieved national coverage in 2008, data preceding this have been backlogged. People with HIV receive information about the register when entering HIV care and give verbal consent or opt out; participants have the right to exit at any time though this has been rare. All people with HIV in active care at the date of data extraction (April 6, 2023) were included.

In order to capture different modalities of difficult-to-treat HIV, we defined five mutually non-exclusive categories:

1. *Advanced resistance.* High-level or intermediate resistance (as per Stanford HIV database [11]) to ≥ 2 antiretrovirals within ≥ 2 drug classes.
2. *4-drug regimen (4DR).* Currently on a 4DR (boosters not counted), having had 2-drug or 3-drug regimen in the past.
3. *Salvage therapy.* Currently on ibalizumab, fostemsavir, enfuvirtide, maraviroc, etravirine, dolutegravir BID (twice daily), or boosted darunavir BID (irrespective of why this regimen was chosen).
4. *Recent virologic failure.* Viral load ≥ 200 copies/mL on 2 measurements (at least 3 months apart) within the past 12 months in a patient on ART for ≥ 5 years.
5. *≥ 2 switches following failure.* Failed virologically ≥ 2 consecutive regimens (≥ 50 copies/mL followed by regimen switch within 6 months) on or after January 1, 2008.

We compared characteristics between participants classified as having difficult-to-treat HIV (belonging to at least one of the five categories) with non-difficult using Mann-Whitney *U* test for continuous variables and Pearson’s *χ*^2^ test for categorical variables. To visualize the number of people in each category and their intersections (since one individual could belong to several categories), we used a Venn diagram. Lastly, we compared treatment outcomes between participants classified as having difficult versus non-difficult-to-treat HIV. For this analysis, we excluded people starting ART < 6 months ago and focused on three different outcomes: virologic suppression, defined as the last available HIV RNA < 50 copies/mL; satisfied with physical health, defined as ≥ 5 on a Likert scale 1–6 (corresponding to the answers “satisfied” or “very satisfied”) in the self-reported health questionnaire; and satisfied with psychological health, defined as ≥ 5 on a similar scale. The health questionnaire has been validated and described previously [12]. The satisfaction Likert scale was dichotomized in accordance with previous studies on InfCareHIV [13]. Since people in the category “Recent virologic failure” by definition have incomplete virologic suppression, we performed a sensitivity analysis excluding this category. To explore if differences in treatment outcomes could be explained by different distributions of baseline characteristics, we also fitted three separate logistic regression models for the three outcomes, including adjustment for sex (man/woman), current age (on a linear scale), and risk group (heterosexual/men who have sex with men/injecting drug use/other).

## Results

Of the currently active individuals in InfCareHIV in April 2023 (*n* = 8531), 799 (9%) met at least one of the definitions of difficult-to-treat HIV. The most frequent category was “≥ 2 switches following failure” (6%), followed by “Advanced resistance” (2%) and “Salvage therapy” (2%). Participants in the difficult-to-treat group were older (median 53 years vs. 51 years; *p* < 0.001), diagnosed earlier (median year 2003 vs. 2009; *p* < 0.001), had lower CD4 nadir (median 140 cells/µL vs. 246 cells/µL; *p* < 0.001), and had a higher first viral load (median 4.7 log_10_ copies/mL vs. 4.4 log_10_ copies/mL; *p* < 0.001) compared with the rest of the cohort. The proportions of people born in Sweden were similar in both groups (31% among people with difficult-to-treat HIV, compared with 33%; *p* = 0.22). Women (assigned sex at birth) were overrepresented among people classified as having difficult-to-treat HIV (45%, compared with 39% among non-difficult; *p* = 0.002), especially in the categories “Recent virologic failure” and “≥ 2 switches following failure”. In the entire cohort, 67% had ≥ 1 recorded resistance test, meaning that the proportion of advanced resistance among those with available resistance data was 188/5726 (3%). The proportion who reported perfect adherence during the last week was 82% among people with difficult-to-treat HIV and 88% among non-difficult (*p* < 0.001); self-reported adherence during the last 24 months was not available for 52% of the cohort, however (Table 1).

**Table 1:** Characteristics of participants with difficult-to-treat HIV, compared with non-difficult (*N* = 8531)

	**Non-difficult-to-treat HIV**	**Difficult-to-treat HIV** ***n*=799 (9%)**				
		**Advanced resistance**	**4DR**	**Salvage therapy**	**Recent virologic failure**	**≥2 switches following failure**
	*n*=7732 (91%)	*n*=188 (2%)	*n*=51 (1%)	*n*=141 (2%)	*n*=59 (1%)	*n*=519 (6%)
Assigned sex at birth						
Man	4698 (61%)	121 (64%)	34 (67%)	85 (60%)	26 (44%)	268 (52%)
Woman	3014 (39%)	67 (36%)	17 (33%)	56 (40%)	33 (56%)	251 (48%)
Missing	20 (0%)	0	0	0	0	0
Current age [year]	51 (42-59)	59 (51-64)	57 (49-61)	58 (50-65)	49 (40-57)	52 (43-59)
Missing	4 (0%)	0	0	0	0	0
Current age < 18 years^a^	89 (1%)	12 (2%)				
Year of first positive HIV test	2009 (2002-2015)	1993 (1990-2003)	1999 (1993-2008)	1996 (1992-2007)	2005 (1998-2010)	2004 (1997-2010)
Missing	140 (2%)	1 (1%)	0	0	1 (2%)	2 (0%)
HIV diagnosis before January 1, 1995	840 (11%)	103 (55%)	15 (29%)	59 (42%)	10 (17%)	90 (17%)
First CD4 count [cells/µL]	370 (200-570)	295 (140-490)	260 (130-490)	230 (140-440)	330 (200-560)	330 (175-520)
Nadir CD4 count [cells/µL]	246 (138-390)	100 (30-190)	130 (40-260)	100 (50-220)	130 (30-230)	160 (70-260)
Missing	29 (0%)	0	0	0	0	0
First HIV RNA [log_10_ copies/mL]	4.4 (2.8-5.1)	4.8 (3.7-5.2)	4.8 (3.6-5.4)	4.9 (3.7-5.6)	4.5 (3.3-5.1)	4.7 (3.8-5.3)
Missing	12 (0%)	0	0	0	0	0
HIV risk group^a^						
Heterosexual	3913 (51%)	430 (54%)				
MSM	2474 (32%)	227 (28%)				
IDU	317 (4%)	31 (4%)				
Other	905 (12%)	108 (14%)				
Missing	123 (2%)	3 (0%)				
Born in Sweden	2567 (33%)	78 (41%)	20 (39%)	58 (41%)	14 (24%)	133 (26%)
Country of birth missing	90 (1%)	1 (1%)	0	1 (1%)	0	1 (0%)
Current ART regimen						
Includes NNRTI	1741 (23%)	47 (25%)	11 (22%)	41 (29%)	8 (14%)	59 (11%)
Includes PI	597 (8%)	104 (55%)	41 (80%)	102 (72%)	13 (22%)	110 (21%)
Includes INSTI	5441 (70%)	160 (85%)	43 (84%)	94 (67%)	42 (71%)	421 (81%)
Includes PI + INSTI	68 (1%)	83 (44%)	34 (67%)	63 (45%)	0	49 (9%)
Has ≥1 resistance test	4999 (65%)	188 (100%)	45 (88%)	117 (83%)	56 (95%)	474 (91%)
Number of missed doses during the last week^a^ (% among those who completed the questionnaire)						
0	3248 (88%)	309 (82%)				
1-2	382 (10%)	54 (14%)				
≥ 3	47 (1%)	14 (4%)				
No completed adherence questionnaire during the last 24 months	4055 (52%)	422 (53%)				

Results are n (%) or median (interquartile range)

*Abbreviations*: *4DR* 4-drug regimen, *ART* Antiretroviral therapy, *IDU* Injecting drug use, *INSTI* Integrase strand transfer inhibitor, *MSM* Men who have sex with men, *NNRTI* Non-nucleoside reverse transcriptase inhibitor, *PI* Protease inhibitor

^a^Results for current age < 18 years, HIV risk group, and ART adherence are aggregated for all people with difficult-to-treat HIV to preserve anonymity, since some combinations contain few individuals

Among the 141 individuals in the category “Salvage therapy”, the most common drug was BID darunavir (*n* = 90), followed by etravirine (*n* = 39) and BID dolutegravir (*n* = 35). The remaining regimens had 5 or less individuals and are not presented to preserve anonymity. The median number of regimen switches was 4 (interquartile range, 3–5) in the category “≥ 2 switches following failure”.

**Fig. 1:**
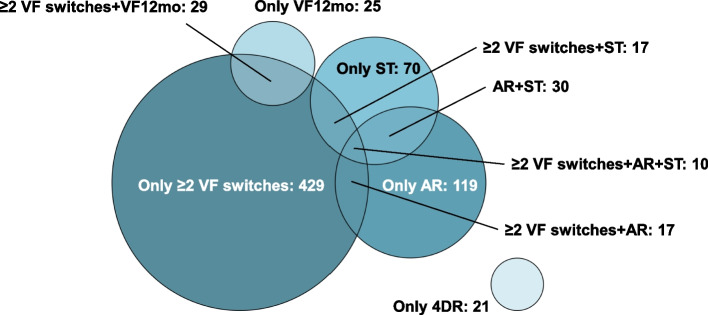
Venn diagram of the number of individuals in the 5 categories of difficult-to-treat HIV. Sets with less than 10 individuals are not shown. “≥ 2 VF switches” denotes people who switched therapy ≥ 2 times in relation to virologic failure, “VF12mo” people with virologic failure within the past 12 months, “AR” people with advanced resistance”, “ST” people currently on salvage therapy, and “4DR” people with 4-drug regimens

We present the intersections between the categories in Fig. 1 (sets with < 10 individuals are not shown). The most common patterns of difficult-to-treat HIV were “≥ 2 switches following failure”, followed by “Advanced resistance”, “Salvage therapy”, and “Advanced resistance + salvage therapy”. For the category “Recent virologic failure”, most people also met the criteria for “≥ 2 switches following failure”. Of people with salvage therapy, only 45 (32%) met criteria for “Advanced resistance”; conversely, 52/188 (28%) of those with advanced resistance received salvage therapy and/or 4DR.

**Fig. 2:**
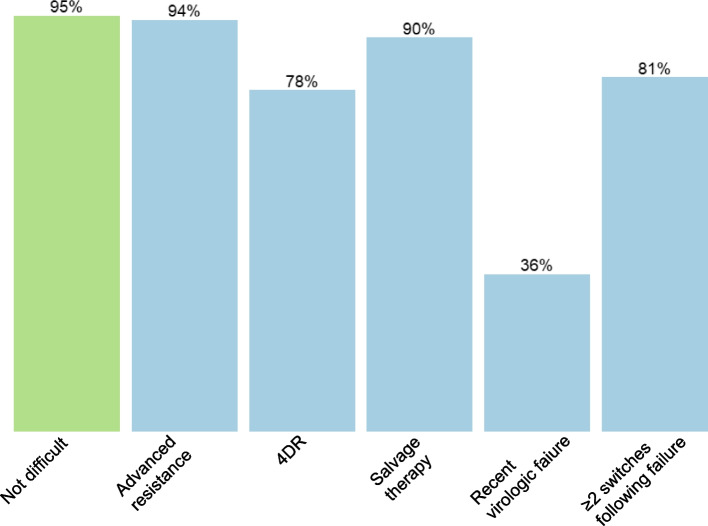
Rates of virologic suppression in the last available viral load measurements among people with difficult vs. non-difficult-to-treat HIV (*n* = 8531). People starting ART < 6 months ago are excluded from this analysis and suppression is defined as HIV RNA in plasma < 50 copies/mL. Viral suppression is assessed in people with a recorded HIV RNA during the last 12 months. Non-difficult-to-treat HIV is shown in green, while the categories of difficult-to-treat HIV are shown in blue

People with difficult-to-treat HIV had a statistically significant lower proportion of viral suppression compared with non-difficult (84% vs. 95%; *p* < 0.001). The suppression rate was highest for the category “Advanced resistance” (94%), followed by “Salvage therapy” (90%), “≥ 2 switches following failure” (81%), “4DR” (78%), and lowest for “Recent virologic failure” (36%) (Fig. 2). When excluding people in the category “Recent virologic failure”, the overall difference in viral suppression rates between people with difficult and non-difficult-to-treat HIV was smaller but still statistically significant (86% vs. 95%; *p* < 0.001). In the subset who had completed the health questionnaire the last 24 months (*n* = 4121), the proportions of participants who were satisfied with their physical health were lower among respondents classified as having difficult-to-treat HIV (55% vs. 63%, *p* = 0.001); this remained statistically significant when adjusting for sex, age, and risk group. There was no difference in psychological health (64% vs. 66%, *p* = 0.29) (Table 2).

**Table 2:** Treatment outcomes among people with difficult-to-treat HIV compared with non-difficult

	**Difficult-to-treat HIV**	**Non-difficult-to-treat HIV**	**Odds ratio (95% CI)**	**Adjusted odds ratio**^a^ **(95% CI)**
Viral suppression < 50 copies/mL (*n* = 8136)	653 (84%)	6991 (95%)	0.29 (0.23-0.36)	0.28 (0.22-0.35)
Satisfied with physical health (*n* = 4121)	212 (55%)	2367 (63%)	0.70 (0.56-0.86)	0.74 (0.60-0.92)
Satisfied with psychological health (*n* = 4116)	247 (64%)	2473 (66%)	0.89 (0.72-1.1)	0.88 (0.71-1.1)

People starting ART < 6 months ago are excluded from this analysis. Viral suppression is assessed in participants with a recorded HIV RNA during the last 12 months. Physical and psychological health was compared in a subset that completed the health questionnaire sometime during the last 24 months

*Abbreviation*: *CI* Confidence interval

^a^Adjusted for sex, age, and risk group

## Discussion

In this study, we estimated the prevalence of difficult-to-treat HIV from resistance data, current antiretroviral regimen, and history of virologic failure and found that approximately 9% of the currently active HIV cohort in Sweden have difficult-to-treat HIV. Importantly, viral suppression rates are relatively high even in this challenging group (84%), but they are lower than the UNAIDS target 95%, which is reached in the Swedish cohort as a whole [3, 4]. Most people classified as having difficult-to-treat HIV in our dataset only met criteria for “≥ 2 switches following failure” (54%). Even though participants in this category have experienced multiple episodes of virologic failure and have been exposed to a median of five ART regimens, we consider this category as the least worrisome as they are currently virologically suppressed on another regimen without having developed advanced resistance.

To our knowledge, no previous study has estimated the prevalence of difficult-to-treat HIV including both HTE and people with recent or repeated failure. Direct comparisons of overall prevalence with other studies are thus not possible, but we can use our different categories to get comparable estimates. Recent studies from the US and Puerto Rico defining HTE based on current regimen have reported 6% and 16% HTE, respectively [5, 14]; this can be compared with 2% (180 individuals) of our cohort belonging to the categories “4DR” and/or “Salvage therapy”. ART criteria in these studies are similar, but not identical to ours. For instance, 4DR was not a criterion for HTE in these studies, but any combination of one INSTI and one PI was. Yet, this does not explain the lower occurrence of HTE in our cohort, since adding the 68 individuals classified as non-difficult but receiving PI + INSTI to the 180 receiving regimens associated with HTE only amounts to 248 (3% of the entire cohort). Other plausible explanations include geographical differences and decreasing prevalence of HTE people with HIV over time (our estimate is from 2023, compared with 2016 in Hsu et al. [5] and 2014–2018 in Priest et al. [14]). When the definition of ART regimens indicative of HTE was expanded to include prior exposure to ≥ 3 core agent classes in Hsu et al. [5], 9% were HTE; this can be compared with 655/8531 (8%) of our cohort belonging to at least one of the categories “4DR”, “Salvage therapy”, and “≥ 2 switches following failure”.

Other studies have defined HTE based on the degree of antiretroviral resistance. The prevalence of advanced resistance where remaining treatment options are limited have decreased significantly the last 20 years, following the introduction of new effective antiretrovirals [15, 16]. In a large European cohort with data from 2010–2016, 1508 (10%) were classified as HTE based on genotypic resistance data, their definition being ≤ 2 drug classes with at least one active agent remaining [6]. This can be compared with 2% of our cohort having reported resistance to ≥ 2 agents in ≥ 2 classes. Importantly, only 24% of the cohort in Pelchen-Matthews et al. [6] had an available resistance test and for the majority the resistance profile was predicted by a model based on other variables. Compared with this, we have high completeness of resistance data (67% with at least one test) so we have no reason to believe that our lower prevalence of advanced resistance is caused by insufficient testing. An analysis of a US database with people starting a new regimen 2015 or later reported very few cases of resistance to ≥ 2 classes (around 0.04% of the entire database); only 24% of people classified as HTE had at least one resistance test, however, and among those with a recorded test, 8% had advanced resistance [17]. Importantly, for people with advanced resistance in our material (of which 72% were treated with standard regimens not meeting criteria for “Salvage therapy” or “4DR”), the suppression rate was 94%, underscoring that today’s potent antiretrovirals (such as INSTIs with a high barrier to resistance) lead to suppression among a high proportion of those who would have had more difficult-to-treat infection before these drugs.

Like in previous studies on HTE, people with difficult-to-treat HIV in our cohort were older, had an earlier first year of positive HIV test, and had lower CD4 nadir compared with non-difficult [5, 6]. The implementation of early ART for everyone can thus be expected to result in lower incidence of difficult-to-treat HIV in the future, even though late diagnosis remains a challenge [18]. Previous reports have not found a higher risk of HTE among women with HIV, however, and the higher risk of difficult-to-treat HIV that we observed was completely explained by virologic failure rather than treatment experience. Around 39% of people with HIV in Sweden are women, and although men who have sex with men have slightly higher suppression rates than people who have acquired HIV through heterosexual contact, there are no large differences in virologic suppression between transmission routes on a national level [3]. Still, our results point to a higher occurrence of recent virologic failure and ≥ 2 switches following failure among women, suggesting that more focus on this patient group might be motivated. Female gender has been reported to predict poor adherence [19], and also in the Swedish HIV cohort, males had significantly higher odds of perfect adherence [12]. Barriers to adherence among women with HIV have not, to our knowledge, been studied in Sweden. Suggested barriers in other settings include depression and other psychiatric conditions, experience of violence, stigma and discrimination, and caregiving stress [20]. Improving adherence among women with HIV should thus be a priority.

Patient-reported outcome measures provide a first-hand perspective of the perceived health among people with HIV. Use of patient-reported outcomes has been shown to improve clinical care and is also recommended in clinical trials [21]. A health questionnaire was introduced to InfCareHIV in 2011, and it has been validated and shown to identify people at risk of failure or in need of additional assessment [12]. To our knowledge, no previous study has analyzed patient-reported health among people with difficult-to-treat HIV, but the lower satisfaction we observed is in line with the high burden of comorbidity that has been described for HTE people [22]. Of note, the causal direction cannot be established from this cross-sectional observation. Other designs are needed to answer whether difficult-to-treat HIV results in worse perceived health, for instance due to side effects of more complicated ART regimens [13], or worse health leads to difficult-to-treat HIV, by impaired adherence [23], more drug-drug interactions due to comorbidities [24], or other mechanisms.

An important limitation of this work is the low coverage of the health questionnaire. Our results on perceived health and self-reported adherence should thus be interpreted with caution, especially since people who complete the questionnaire likely represent a distinct subset with high involvement in care (non-response bias) [25]. The low number of completed questionnaires also precluded us from analyzing associations between separate sub-categories of difficult-to-treat HIV and patient-reported outcome measures. Moreover, we lack information on medical and psychiatric comorbidities as well as non-ART medication. It should also be noted that the definition of the category “≥ 2 switches following failure” included both switches due to failure and due to other reasons (since the cause of switching can be difficult to delineate in register data). Gender identity has recently been added to the Swedish HIV register, but as of this data extraction, low coverage precludes use of this variable. As for other observational studies, there is a risk of residual confounding for relationships between difficult-to-treat HIV and treatment outcomes. Strengths of this work include complete national coverage, high frequency of resistance testing, and inclusion of patient-reported outcomes for a subset of the cohort.

## Conclusions

We developed a definition of difficult-to-treat HIV that incorporated both HTE and recent or repeated virologic failure. Based on this, we identified 9% of the current Swedish HIV cohort as individuals with potentially challenging-to-treat HIV, but issues with recent failure, advanced resistance, and need for salvage therapies are rare compared with other cohorts. For most of the Swedish HIV cohort, strategies to promote health and ART adherence among people with HIV seems more called for than new therapies for HTE individuals.

## Data Availability

Data cannot be shared publicly due to confidentiality of participants’ information. Data can be made available from InfCareHIV upon reasonable request and after ethical approval from the Swedish Ethical Review Authority. Instructions and contact information can be found at www.infcarehiv.se.

## Abbreviations

4DR: 4-drug regimen
ART: Antiretroviral therapy
BID: Twice daily
HTE: Heavily treatment-experienced
